# Reliable genetic diagnosis of *NCF1* (p47^phox^)-deficient chronic granulomatous disease using high-throughput sequencing

**DOI:** 10.3389/fimmu.2025.1640496

**Published:** 2025-08-18

**Authors:** Amy P. Hsu, Eric Karlins, Justin Lack, T. Joseph Pepper, Karen Lau, Kimberly R. Marshall-Batty, Debra Long Priel, Joie Davis, Danielle L. Fink, Christa S. Zerbe, John I. Gallin, Harry L. Malech, Steven M. Holland, Douglas B. Kuhns

**Affiliations:** ^1^ Immunopathogenesis Section, Laboratory of Clinical Immunology and Microbiology, National Institute of Allergy and Infectious Diseases, National Institutes of Health (NIH), Bethesda, MD, United States; ^2^ Bioinformatics and Computational Biosciences, Office of Cyber Infrastructure and Computational Biology, National Institute of Allergy and Infectious Diseases (NIAID), National Institutes of Health (NIH), Bethesda, MD, United States; ^3^ National Institute of Allergy and Infectious Diseases (NIAID) Collaborative Bioinformatics Resource, National Institute of Allergy and Infectious Diseases (NIAID) National Institutes of Health (NIH), Bethesda, MD, United States; ^4^ Advanced Biomedical Computational Science, Frederick National Laboratory for Cancer Research, Leidos Biomedical Research, Inc., Frederick, MD, United States; ^5^ Department of Mathematics, University of Maryland College Park, College, Park, MD, United States; ^6^ Neutrophil Monitoring Laboratory, Applied/Developmental Research Directorate, Leidos Biomedical Research, Inc, Frederick National Laboratory for Cancer Research, Frederick, MD, United States; ^7^ Laboratory of Clinical Immunology and Microbiology, National Institute of Allergy and Infectious Diseases, National Institutes of Health (NIH), Bethesda, MD, United States; ^8^ Genetic Immunotherapy Section, Laboratory of Clinical Immunology and Microbiology, National Institute of Allergy and Infectious Diseases, National Institutes of Health (NIH), Bethesda, MD, United States

**Keywords:** NCF1, CGD, chronic granulomatous disease (CGD), NGS, ONT long read sequencing, pseudogene, genetic diagnosis

## Abstract

**Introduction:**

Chronic granulomatous disease is caused by mutations in any of the 6 components of the phagocytic NADPH oxidase complex including gp91^phox^, p47^phox^, p22^phox^, p40^phox^, p67^phox^, or EROS. Functional assays include reactive oxygen species (ROS) production, flow cytometry, and immunoblotting for NADPH proteins. The advent of high-throughput sequencing allows genetic diagnosis for all components except *NCF1* (p47^phox^) due to two, nearly identical, pseudogenes (*NCF1B*, *NCF1C*). The majority of NCF1-CGD patients carry a 2-base deletion caused by crossover between *NCF1* and *NCF1B* or *NCF1C*. Currently, NCF1 deficiency is diagnosed functionally: a characteristic DHR with low levels of residual ROS, loss of p47^phox^ on immunoblot, or digital droplet PCR or Gene-scan to enumerate intact (GTGT) or deleted (ΔGT). While this provides patients a clinical CGD diagnosis, for the 20% of NCF1-CGD patients with a non-ΔGT mutation a definitive genetic diagnosis is still lacking.

**Methods:**

We developed a bioinformatic method using existing short or long-read sequencing data from 48 NCF1-CGD patients or carriers.

**Results:**

We identified both ΔGT and non-ΔGT *NCF1* gene mutations. Additionally, we confirm that the presence of ΔGT in *NCF1* is due to pseudogene copy into the *NCF1* locus. We compare *NCF1* sequence from NCF1-CGD patients to cohorts of non-NCF1-CGD and healthy controls (1000Genomes), demonstrating pseudogene replacement of *NCF1* in NCF1-CGD as well as the reciprocal replacement of *NCF1B* or *NCF1C* by *NCF1* in some healthy controls.

**Discussion:**

With this method, reanalysis of existing sequence data may provide genetic diagnosis to NCF1-CGD patients. This technique may be modified for other diagnostically relevant pseudogenes.

## Introduction

Chronic granulomatous disease (CGD) is caused by mutations in any of the 6 subunits of the phagocyte nicotinamide adenine dinucleotide phosphate (NADPH) oxidase (phox). Patients frequently, but not always, present as young children with recurrent bacterial and/or fungal infections. The majority (68%) of patients in Western countries carry mutations in the X-linked *CYBB*, encoding gp91^phox^ (1, 2), while bi-allelic *NCF1* mutations, encoding p47^phox^, occur in 25% (2), although this number is significantly larger in regions with high levels of consanguinity (3, 4). Access to large scale diagnostic sequencing, including targeted panel, whole exome, and whole genome, has enabled identification of mutations for the majority of CGD patients. However, due to the presence of two highly homologous pseudogenes (*NCF1B*, *NCF1C*, together, *ΨNCF1*), identification of specific *NCF1* mutations remains challenging. Given the 99.5% homology, sequence reads fail to uniquely align to the reference genome, causing lack of coverage and inability to make variant calls. Additionally, the presence of the two pseudogenes essentially creates 6 copies of *NCF1*, limiting reliability of Sanger sequencing.

**Figure 1:**
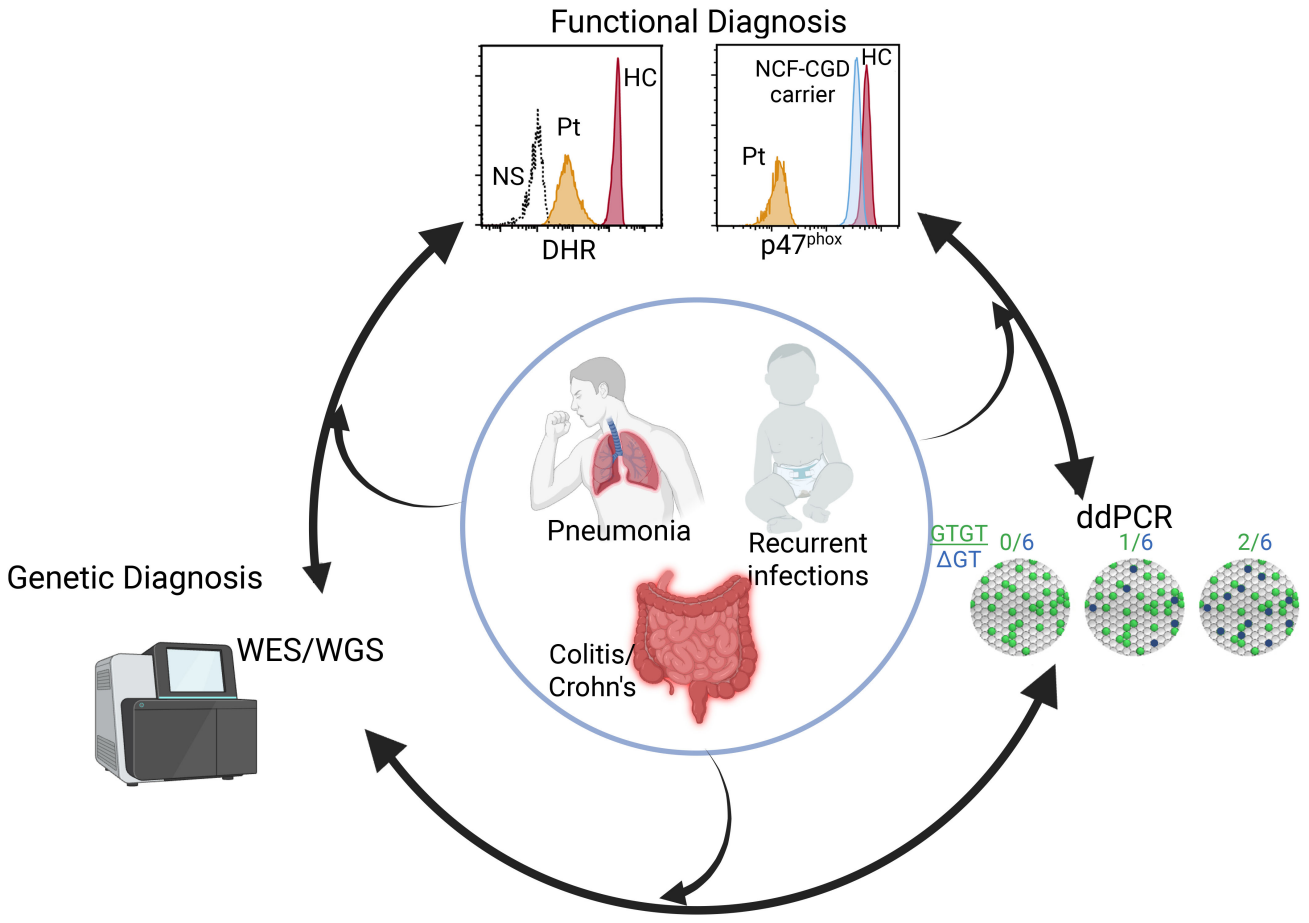
Diagnostics of NCF1-CGD. Patients (inner circle) presenting with disparate phenotypes may enter the diagnostic pipeline at different points including functional studies, sequencing, or for those with a family history, ddPCR or Gene-scan. Identification of genomic variant requires functional assessment while abnormal neutrophil functional studies leads to sequencing and a genetic diagnosis. Figure created in BioRender.

Patients suspected to have CGD undergo functional testing including flow cytometry or immunoblotting for NADPH oxidase proteins, ROS production by dihydrorhodamine (DHR), cytochrome C reduction, and/or nitroblue tetrazolium (NBT) reduction assay (**Figure 1**). While some larger hospital settings perform DHR or NBT assays, frequently patient samples are sent to commercial diagnostic laboratories. Comprehensive neutrophil studies encompassing all of these assays are only available in a handful of dedicated research laboratories. Reduced or absent DHR indicates a defective NADPH complex; loss of one component protein, demonstrated by flow cytometry or immunoblot, may indicate the mutated subunit, however none of these assays reveals underlying genetics. Whole exome, whole genome, or targeted capture panel sequencing provides genetic diagnosis for the other 5 NADPH oxidase components but not *NCF1*. *NCF1* genetics are currently limited to enumeration of pseudogene and *NCF1* copy number by Gene-scan (5) or droplet digital PCR (ddPCR) (2), or Sanger sequencing with anchored primers (6).

There are several nucleotides differentiating *NCF1*, *NCF1B*, and *NCF1C*, with the crucial difference being a two-base deletion at the start of exon 2 (ΔGT) in *ΨNCF1*, resulting in frameshift and premature termination (7). Recombination between *NCF1* and *NCF1B* or *NCF1C* has been posited as the cause of the most common genetic variation, incorporation of ΔGT in *NCF1 (* 8). Currently, the Gene-scan assay (5) is the most common method for ΔGT identification; recently, assays to determine the ratio of intact GTGT vs *NCF1/NCF1B/NCF1C* copy number by ddPCR (2) or restriction fragment length polymorphism (9) were reported, which provide a ΔGT genetic diagnosis. All three methods are research assays not commercially or commonly available. CGD patients lacking NCF1 protein, yet carrying 1 or 2 intact GTGT alleles, remain without a genetic diagnosis. Herein we describe a bioinformatic pipeline to enable re-analysis of short- and long-read sequence data to identify non-ΔGT *NCF1* mutations in NCF1-CGD patients and NCF1-CGD carriers previously lacking genetic diagnoses. This analysis supports the crossover theory between pseudogene and *NCF1* and demonstrates multiple discrete recombinations indicating recurring events. Using 1000 Genomes (1000G) data as controls, we also demonstrate the presence of reciprocal replacement of pseudogenes by *NCF1*.

## Methods

### Patient cohort

Patients with previously diagnosed chronic granulomatous disease (CGD) (n = 45) and first-degree relatives (n = 3) followed at, or referred to, the National Institutes of Health were included in the study after being consented to IRB-approved protocols NCT00001355, NCT00404560, NCT00001467, or NCT00128973. Routine functional analysis included DHR assay for ROS production, immunoblot and/or flow cytometry to determine the presence of specific NADPH oxidase component proteins, and ddPCR for quantification of ΔGT as previously published (2). Illumina short-read whole exome (n = 21) or whole genome (n = 21) sequencing was performed at commercial laboratories (Johns Hopkins Genomics, Baltimore, MD; Baylor College of Medicine Human Genome Sequencing Center, Houston, TX). Oxford Nanopore long-read whole genome sequencing (n = 6) was performed at Johns Hopkins Genomics, Genetics Resource Core Facility, Baltimore, MD.

Non NCF1-CGD patients included 48 patients with X-linked *CYBB* mutations (including 4 skewed female patients), 4 bi-allelic *CYBA*, and 1 bi-allelic *NCF2*, recruited under the same protocols.

### Reference sequences

NCBI reference sequences for *NCF1* (NM_00236.7; NP_000256.4), *NCF1B* (NR_003186), and *NCF1C* (NR_003187) were aligned using Sequencher (GeneCodes, Ann Arbor, MI).

### Bioinformatic pipeline

To effectively map sequencing reads to this region we created an hg38 reference fasta file by masking the pseudogene sequence (chr7:73220639–73235945 and chr7:75156639-75172044) with “N”s using “bedtools maskfasta” (10, 11). Reads were mapped to our masked-reference using bwa-mem (https://bio-bwa.sourceforge.net/bwa.shtml). Variant calling was performed using GATK Best Practices (12), altering the ploidy to 6 to adjust for the tripling of reads in our region of interest. A custom script (https://github.com/niaid/NCF1_variant_calling), adapted from Almeida de Jesus, et al (13), was used to emit putative variant sites when 2 or more reads with alternate alleles were present, regardless of read balance. These sites were then fed to GATK for genotyping. The result of these methods is a list of predicted variants, most of which we were unable to discover using standard methods. We also calculate the alternate allele balance (AltAB) defined as the frequency of variant allele reads versus total read depth from *NCF1* plus *NCF1B/NCF1C* at any given nucleotide. While AltAB is equivalent to variant allele frequency (VAF) used for high throughput sequencing data, given the 6 alleles present in the *NCF1/NCF1B/NCF1C* locus, we use AltAB to distinguish from normal, heterozygous loci.

### 1000 Genome reference table

Variants identified using publicly accessible 1000G short read sequence data (https://ftp.sra.ebi.ac.uk/vol1/run/) were parsed by ancestry to provide a reference dataset of masked *NCF1* variant frequency in a healthy control population (**Supplementary Table 1**, 1000_Genomes_NCF1_variants.xlsx).

### PCR for P5/P6 large deletion

Primers were selected from outside the deleted region and used to amplify DNA from P5, P6, and the father of P6 (P6-F). Primers used were NCF1del 169F 5’- AAGATAAACCCAAACTAAGGGACATTCTACAAGG– 3’ and NCF1del 5960R 5’ – ATTTTATTTTGAGATGGAGTTTTGTCCTTGTTGC – 3’. Amplification was performed in 15 μl reactions using Platinum Taq HiFi (ThermoFisher) and cycling conditions 95°C 3 min, (95°C 20 sec, 62.3°C 10 sec, 68°C 30 sec) x35, 68°C 3 min; product was visualized on 1.2% agarose gel.

## Results

### Cohort description

Patients were referred to the NIH for clinical evaluation or functional testing for suspected chronic granulomatous disease. All patients (n=48; 45 NCF1-CGD plus 3 NCF1-carriers) (**Table 1**) were characterized by functional assays including flow cytometric DHR, immunoblots and/or flow cytometry for gp91^phox^ (*CYBB*), p22^phox^ (*CYBA*), p47^phox^ (*NCF1*), p67^phox^ (*NCF2*), and p40^phox^ (*NCF4*). All NCF1-CGD patients had diminished PMA-induced neutrophil superoxide production and DHR (**Supplementary Table 2**) and undetectable p47^phox^ by immunoblot or flow cytometry. Frequency of ΔGT for each individual was performed using ddPCR (**Table 1**). Genetic sequencing included short-read whole exome (WES) (n=21) or whole genome (WGS) (n=21) or long-read WGS (n=3 NCF1-CGD patients and 3 NCF1 carriers).

**Table 1:** NCF1-CGD patients.

Patient ID	Disease	GTGT copies^%^	Read depth	Alt call	AltAB	Break point	cDNA mutation	Protein mutation	CADD	Seq platform
P1^A^	NCF1-CGD	2	113	24	0.2123	none	c.72 + 1G>A	splice	33.0	WES
			1256	198	0.1576		c.125G>A	p.R42Q	33.0	WES
P2	NCF1-CGD	1	97	78	0.8041	ex 2-4, intron 5	c.75_76del	p.Tyr26fs	38.0	WGS
			140	18	0.1286		c.839T>C	p.L280P	33.0	WGS
P3	NCF1-CGD	1	131	116	0.8855	ex 2-4, intron 5	c.75_76del	p.Tyr26fs	38.0	WGS
			161	20	0.1242		c.892_905 + 11del	p.A298fs	nd	WGS
P4	NCF1-CGD	1	99	84	0.8485	ex 2-4, intron 5	c.75_76del	p.Tyr26fs	38.0	WGS
			163	27	0.1656		c.574G>A	p.G192S (splice)	34.0	WGS
P5^B^	NCF1-CGD	1	1287	1063	0.8260	ex 2-4, intron 5	c.75_76del;	p.Tyr26fs;	38.0	WES
							NC_000007.14: 74770624-74776017del	p.1M?	nd	PCR
P6^B^	NCF1-CGD	1	90	69	0.7667		c.75_76del;	p.Tyr26fs	38.0	ONT
			69	17	0.2464		NC_000007.14: 74770624-74776017del	p.1M?	nd	ONT
P7	NCF1-CGD	1	141	111	0.7872		c.75_76del;	p.Tyr26fs	38.0	ONT
			131	15	0.1145		c.574G>A	p.G192S (splice)	34.0	ONT
P8	NCF1-CGD	2	37	5	0.1351		c.72 + 3G>T	splice	21.6	ONT
			33	9	0.2727		c.579G>A^	p.W193*	37.0	ONT
			38	10	0.2632		c.500A>C^	p.Y167S	23.1	ONT
P9^A^	NCF1 carrier	2	124	14	0.1129		c.125G>A	p.R42Q	33.0	ONT
P10	NCF1 carrier	2	91	16	0.1758		c.574G>A	p.G192S (splice)	34.0	ONT
P11	presumed NCF1 carrier	3					No variant detected			ONT
P12^C^	NCF1-CGD	0	113	113	1	none	hom c.75_76del	p.Tyr26fs	38.0	WGS
P13^C^	NCF1-CGD	0	1369	1365	0.9971	none	hom c.75_76del	p.Tyr26fs	38.0	WES
P14	NCF1-CGD	0	442	442	1	none	hom c.75_76del	p.Tyr26fs	38.0	WES
P15	NCF1-CGD	0	574	572	0.9965	none	hom c.75_76del	p.Tyr26fs	38.0	WES
P16	NCF1-CGD	0	311	310	0.9968	ex 2-4	hom c.75_76del	p.Tyr26fs	38.0	WES
P17	NCF1-CGD	0	775	775	1	intron 5	hom c.75_76del	p.Tyr26fs	38.0	WES
P18^D^	NCF1-CGD	0	1037	1037	1	none	hom c.75_76del	p.Tyr26fs	38.0	WES
P19^D^	NCF1-CGD	0	1037	1037	1	none	hom c.75_76del	p.Tyr26fs	38.0	WES
P20^E^	NCF1-CGD	0	659	659	1	none	hom c.75_76del	p.Tyr26fs	38.0	WES
P21^E^	NCF1-CGD	0	732	732	1	intron 5	hom c.75_76del	p.Tyr26fs	38.0	WES
P22	NCF1-CGD	0	1047	1047	1	none	hom c.75_76del	p.Tyr26fs	38.0	WES
P23	NCF1-CGD	0	479	478	0.9979	none	hom c.75_76del	p.Tyr26fs	38.0	WES
P24	NCF1-CGD	0	717	717	1	none	hom c.75_76del	p.Tyr26fs	38.0	WES
P25	NCF1-CGD	0	545	543	0.9963	intron 8	hom c.75_76del	p.Tyr26fs	38.0	WES
P26^F^	NCF1-CGD	0	1366	1366	1	none	hom c.75_76del	p.Tyr26fs	38.0	WES
P27^F^	NCF1-CGD	0	118	118	1	none	hom c.75_76del	p.Tyr26fs	38.0	WGS
P28	NCF1-CGD	0	531	528	0.9944	intron 5	hom c.75_76del	p.Tyr26fs	38.0	WES
P29	NCF1-CGD	0	73	73	1	none	hom c.75_76del	p.Tyr26fs	38.0	WGS
P30	NCF1-CGD	0	129	129	1	none	hom c.75_76del	p.Tyr26fs	38.0	WGS
P31	NCF1-CGD	0	1123	1123	1	intron 5	hom c.75_76del	p.Tyr26fs	38.0	WES
P32	NCF1-CGD	0	594	594	1	none	hom c.75_76del	p.Tyr26fs	38.0	WES
P33	NCF1-CGD	0	129	129	1	none	hom c.75_76del	p.Tyr26fs	38.0	WGS
P34	NCF1-CGD	0	902	902	1	none	hom c.75_76del	p.Tyr26fs	38.0	WES
P35	NCF1-CGD	0	1540	1540	1	none	hom c.75_76del	p.Tyr26fs	38.0	WES
P36	NCF1-CGD	0	122	122	1	undet	hom c.75_76del	p.Tyr26fs	38.0	WGS
P37	NCF1-CGD	0	130	130	1	none	hom c.75_76del	p.Tyr26fs	38.0	WGS
P38	NCF1-CGD	0	113	113	1	none	hom c.75_76del	p.Tyr26fs	38.0	WGS
P39	NCF1-CGD	0	119	119	1	none	hom c.75_76del	p.Tyr26fs	38.0	WGS
P40	NCF1-CGD	0	83	83	1	none	hom c.75_76del	p.Tyr26fs	38.0	WGS
P41	NCF1-CGD	0	119	119	1	none	hom c.75_76del	p.Tyr26fs	38.0	WGS
P42	NCF1-CGD	0	99	99	1	intron 4-5	hom c.75_76del	p.Tyr26fs	38.0	WGS
P43	NCF1-CGD	0	93	93	1	none	hom c.75_76del	p.Tyr26fs	38.0	WGS
P44	NCF1-CGD	0	81	81	1	intron 5	hom c.75_76del	p.Tyr26fs	38.0	WGS
P45	NCF1-CGD	0	100	100	1	intron 5	hom c.75_76del	p.Tyr26fs	38.0	WGS
P46	NCF1-CGD	0	87	87	1	none	hom c.75_76del	p.Tyr26fs	38.0	WGS
P47	NCF1-CGD	0	83	83	1	none	hom c.75_76del	p.Tyr26fs	38.0	WGS
P48	NCF1-CGD	0	85	85	1	none	hom c.75_76del	p.Tyr26fs	38.0	WGS

^%^ - Number of GTGT copies as determined by ddPCR; Related individuals denoted by matching superscript letters (A, B, C, D, E, F); ^ - Allelic variants in P8; hom, homozygous; WES, whole exome short-read sequencing; WGS, whole genome short-read sequencing; ONT, Oxford Nanopore Technologies long-read whole genome sequencing; undet, undetermined.

Laboratory testing among the NCF1-CGD cohort resembled previously reported patients (2, 14) with all patients displaying residual ROS after PMA stimulation and absent p47^phox^ protein (2). Among NCF1-CGD patients, ddPCR results demonstrated 37/45 (82.2%) had no intact GTGT alleles, 6/45 (13.3%) had 1 GTGT allele, while 2/45 (4.4%) had 2 intact GTGT alleles.

### Assignment of genotype from pseudogene masking

**Figure 2:**
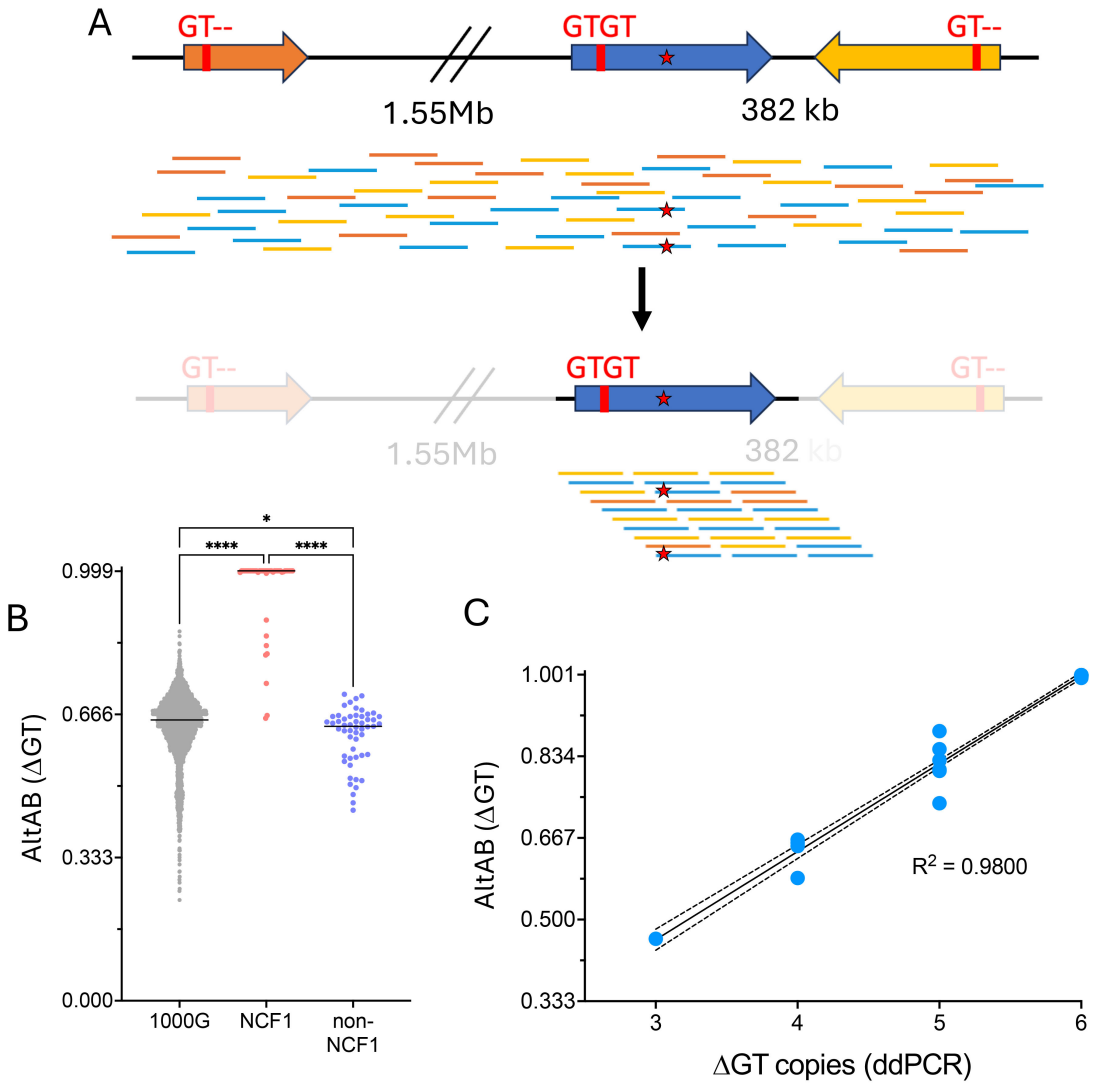
Utilization of masked genome to identify the common NCF1 ΔGT mutation. **(A)** *NCF1* (blue) is flanked by 2 pseudogenes, *NCF1B* upstream (orange) and *NCF1C* downstream in reverse orientation (yellow). Both *NCF1B* and *NCF1C* have a deletion at the start of exon 2, noted “GT–” while NCF1 has intact, GTGT sequence. Short read, unmapped sequences for *NCF1/NCF1B/NCF1C* are shown scattered across the loci (top). Mutation within NCF1 is denoted by red star. Using a masked genome to prevent alignment to *NCF1B* or *NCF1C* (bottom), all reads align to *NCF1* allowing calling of non-ΔGT mutation. **(B)** Variant allele frequency (AltAB) for ΔGT from 1000G, NCF1-CGD, and non-NCF1-CGD cohorts. (****P<0.0001, *P=0.0211 ANOVA with Kruskal-Wallis test for multiple comparisons) **(C)** Simple linear regression curve ddPCR determined ΔGT copies compared to AltAB of ΔGT variant per individual.

The presence of *NCF1B*/*NCF1C* (*ΨNCF1*) prevents unique alignment of sequencing reads. Therefore, we developed a bioinformatic pipeline to mask *ΨNCF1*, thereby aligning all sequence reads to *NCF1* (**Figure 2A**). Utilizing this pipeline we then performed variant calling using standard methods. Adjusting the ploidy parameter from 2 to 6 allowed identification of variants from *NCF1* reference sequence. For each variant, the ratio of variant calls to read depth was used to establish the alternate allele balance (AltAB) from *NCF1B* and *NCF1C*. Given that 3 unique autosomal genomic regions were included, AltAB increments approximate 1/6, with variants occurring in both *ΨNCF1* loci having AltAB ~0.66 and those occurring in only *NCF1B* or *NCF1C* having AltAB ~0.33. We then used AltAB of ΔGT to compare 1000G, NCF1, and non-NCF1 CGD patients. Both 1000G and non-NCF1 patients had median AltAB of 0.66 indicating 4 copies of ΔGT from *ΨNCF1* and two intact *NCF1* GTGT alleles; by contrast, NCF1 patients had a median AltAB of 1.0 (indicating no remaining GTGT alleles) with only 11 individuals deviating from that (**Figure 2B**). Since we had previously performed ddPCR to determine ΔGT copies in these patients, we compared AltAB to ddPCR results (**Figure 2C**). Each of the 11 samples with ΔGT AltAB<1 (P1 through P11) was from patients with 1 (n=6) or 2 (n=2) copies of GTGT; also included were 3 *NCF1* mutation carriers, with 2 (n=2) or 3 (n=1) copies of GTGT. Plotting ddPCR-determined GTGT copy number versus AltAB demonstrated concordance between the two methods (R^2^ = 0.980), validating the use of AltAB to detect the most common *NCF1* mutation.

### Variant occurrences in 1000G

Using NCBI reference sequences for the 3 loci we established a variant table, allowing assignment of variants to *NCF1B*, *NCF1C* or both (Ψ*NCF1*) (**Supplementary Table 3**). We then examined the AltAB distribution of these variants among 1000G. *ΨNCF1* variants (**Supplementary Figure 1**) mostly displayed a median AltAB (mAltAB) of 0.66, consistent with 4/6 copies of ΔGT, although the distribution was broad with 3/6 or 5/6 copies not uncommon and several other variants displaying 2/6 copies. For 9 loci, mAltAB was 1.0 indicating incorporation of the variant in *NCF1* or incorrect *NCF1* reference sequence. It is noteworthy that, despite a normal distribution and mAltAB ~0.66, 12 individuals have AltAB=1.0 for c.269G>A encoding p.R90H (**Supplementary Figure 1**, blue).

Examining *NCF1C* variants, in which mAltAB should be ~0.33, 3/14 variants have mAltAB>0.33 with multimodal distribution; an additional 4 variants have mAltAB<0.33, 2 of which have bimodal distributions of 1/6 or 2/6 while the remaining 2 variants have mAltAB=0.26 **(Supplementary Figure 2**). A similar *NCF1B* variant analysis reveals 2/14 variants with mAltAB~0.66, 4/14 variants with multimodal distributions and mAltAB>0.33 (**Supplementary Figure 3**). Collectively, this analysis identifies a subset of variants having frequencies consistent with their presence in one or both pseudogenes (noted in **Supplementary Table 3**) available for further analyses of the locus.

**Figure 3:**
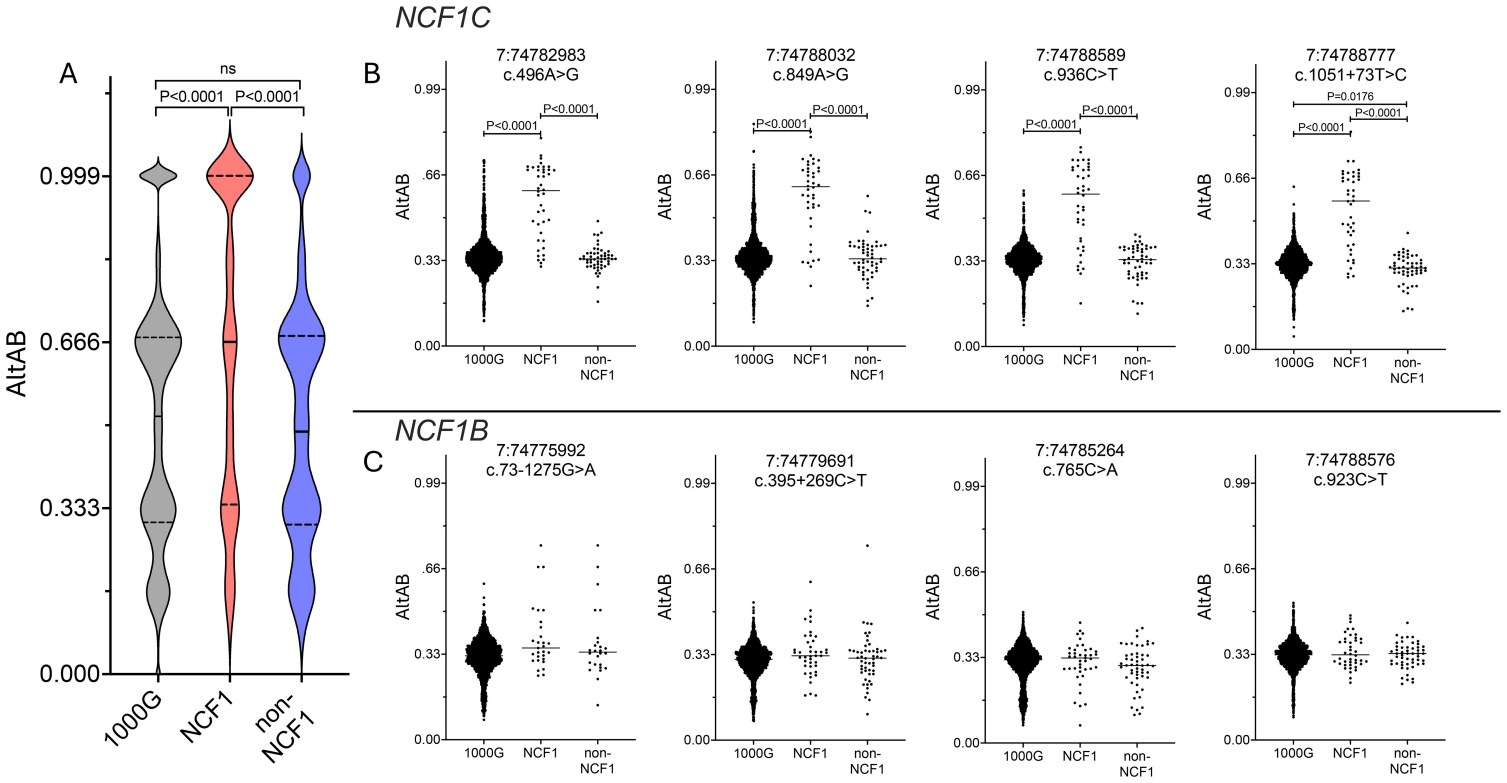
NCF1-CGD patients have more pseudogene copies and these are more frequently NCF1C. **(A)** AltAB across *NCF1* locus for 1000G (grey), NCF1 (red) and non-NCF1 (blue) CGD patients. Violin plots with median (solid line) and quartiles (dotted lines) indicated. Groups compared using Ordinary one-way ANOVA with Tukey’s multiple comparisons test. B and **(C)** AltAB of *NCF1C* **(B)** or *NCF1B* **(C)** specific variants across three cohorts. Groups compared using Ordinary one-way ANOVA with Tukey’s multiple comparisons test. Only comparisons with adjusted P<0.05 are shown.

Using these data, we next examined distribution of AltAB across *NCF1* among NCF1 patients, 1000G, and non-NCF1 CGD patients. It was previously suggested that inclusion of ΔGT arises from meiotic crossover between *NCF1* and one of the pseudogenes (7). Supporting this, NCF1-CGD patients have higher AltAB values throughout the *NCF1* locus compared to 1000G controls and non-NCF1 patients (**Figure 3A**) indicating a higher proportion of *ΨNCF1* variants among patients. NCF1 patients displayed a clear skewing of AltAB with mAltAB=0.66 while both 1000G and non-NCF1 patients had mAltAB of 0.52 and 0.49 respectively. This suggests larger incorporation of Ψ*NCF1* than solely the ΔGT-containing exon 2 (**Figure 2B**). To explore this, we plotted AltAB across 4 variants specific to *NCF1C* or *NCF1B*. NCF1 patients display increased AltAB across *NCF1C* variants compared to 1000G or non-NCF1 patients (**Figure 3B**); by contrast, there is no significant increase in AltAB across *NCF1B* SNPs (**Figure 3C**), although some individual patients have increased *NCF1B* SNP AltAB. Together, these data suggest *NCF1* replacement with *NCF1C* occurs more frequently than with *NCF1B*.

### Mapping *NCF1* loci recombination

**Figure 4:**
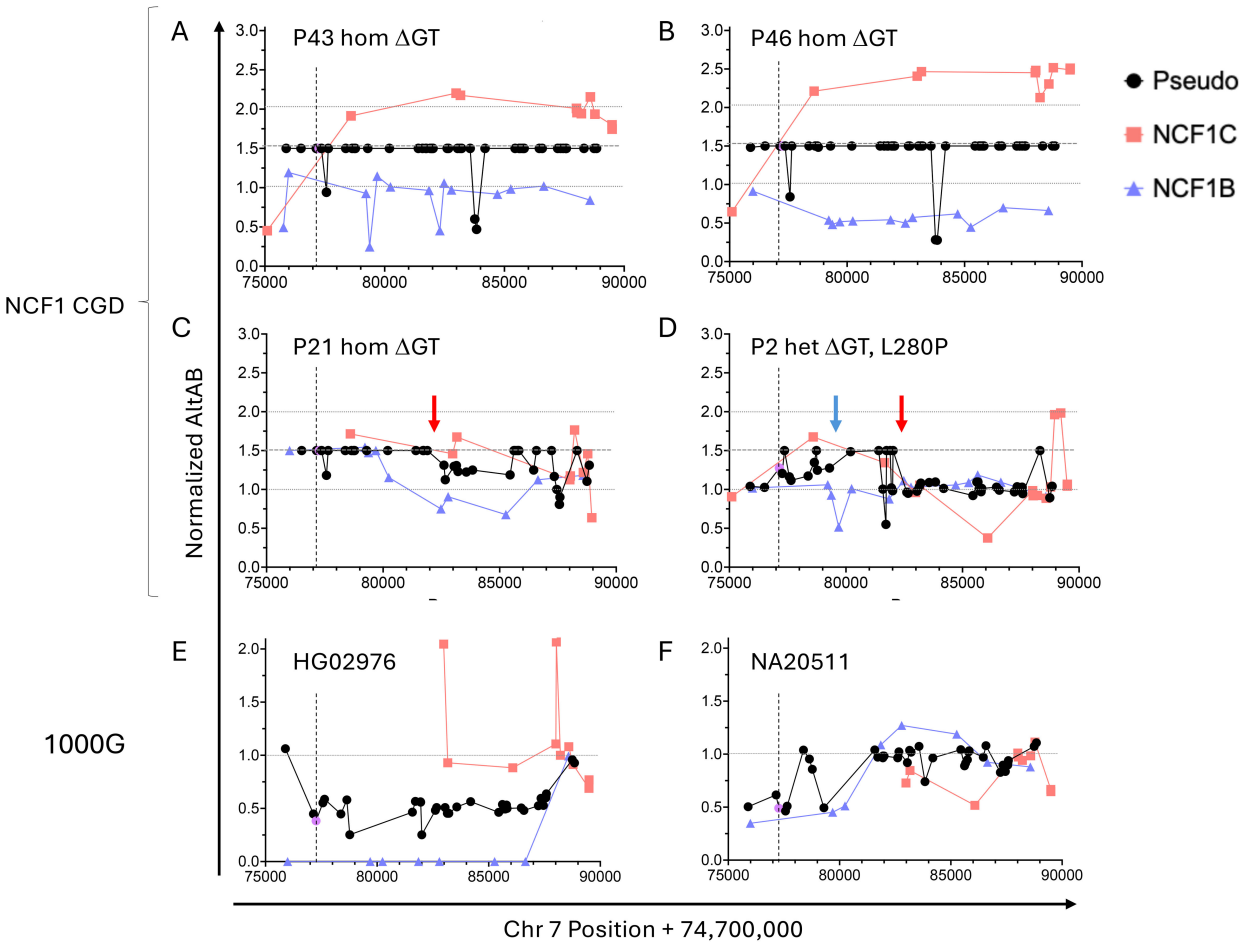
Normalized AltAB shows *NCF1/NCF1B/NCF1C* rearrangement among NCF1-CGD patients and healthy controls. Variants present in both pseudogenes (black) should have AltAB~0.66, variants present in *NCF1C* (red) or *NCF1B* (blue) should have AltAB~0.33; ΔGT is noted in purple. Each patient’s AltAB was normalized (nAltAB) for expected values based on 1000G data. Vertical line denotes location of ΔGT variant. **(A)** P43, homozygous ΔGT, with nAltAB of 1.5 for pseudogene variants while *NCF1C*-specific variants have nAltAB = ~2 indicating *NCF1* replacement by *NCF1C*; *NCF1B* maintains nAltAB~1.0. **(B)** Similar analysis of P17, homozygous ΔGT, nAltAB =1.5 for pseudogene variants, nAltAB for *NCF1C* variants ~2. 5 while nAltAB *NCF1B* variants ~ 0.5 indicating 5 copies of *NCF1C* and only 1 copy of *NCF1B*. **(C)** P25, homozygous ΔGT, with only 5’ end of *NCF1* replaced by pseudogene but normalized AltAB after intron 5 breakpoint (red arrow). **(D)** P2, compound heterozygous for ΔGT and L280P, demonstrating two breakpoints between exons 2-4 (blue arrow) and intron 5 (red arrow). E and **(F)** 1000G controls with nAltAB < 1.0. **(E)** HG02976 has pseudogene nAltAB ~ 0.5 indicating only 2 copies of pseudogene present; nAltAB for *NCF1B* variants = 0 indicating both pseudogene copies present are *NCF1C*. **(F)** NA20511 has pseudogene nAltAB ~ 0.75 indicating loss of 1 pseudogene copy, nAltAB for *NCF1C* ~ 0.5 suggests only 1 copy of *NCF1C*.

These data suggested full or partial replacement of *NCF1* with *NCF1C* in the majority of ΔGT *NCF1* alleles, which we sought to confirm. Using variants demonstrated to have normal distribution among 1000G, we normalized AltAB at each site to 1000G mAltAB and plotted the normalized value across the locus. For patients with no intact GTGT, there would be 6 copies of ΔGT rather than 4, giving a normalized AltAB of 1.5. In the majority of patients (27/37 homozygous ΔGT, 73.0%), we observe normalized AltAB of 1.5 at Ψ*NCF1* SNPs across the locus indicating full replacement of *NCF1* with a pseudogene (**Figures 4A, B**); additionally, 2/4 heterozygous ΔGT patients replaced one copy of *NCF1* with a pseudogene resulting in normalized AltAB of ~1.25. Using the *NCF1B* or *NCF1C* specific variants, we observed cases of full *NCF1* replacement by *NCF1C* as demonstrated by a normalized *NCF1C* AltAB of 2 across the locus indicating 4 copies of *NCF1C* and 2 copies of *NCF1B* (P43, **Figure 4A**). Alternative arrangements are present as well, including replacement of *NCF1* in the setting of 5 copies of *NCF1C* and only 1 *NCF1B* (P46, **Figure 4B**).

Not all patients replaced the full gene, there was a frequent crossover point in intron 5 (**Figures 4C, D** red arrows) corresponding to consecutive *AluJr* and *AluSx1* repeats present in all three loci. Among NCF1-CGD patients with short-read sequencing (n=42), we could identify recurrent breakpoints between exons 2-4 (5/42, 11.9%) (**Figure 4D**, blue arrow) and within intron 5 (10/42, 23.8%); 4 patients within these two groups exhibited both breakpoints (**Figure 4D**), one patient each had breaks between introns 4–5 and within intron 8; one patient was indeterminate due to lack of informative SNPs (**Table 1**). In P21, *NCF1* was replaced by *NCF1C* on one allele and only the 5’ portion of *NCF1B* on the other (**Figure 4C**). Lastly, P2 carried a missense mutation, (c.839T>C, p.L280P) on one allele, and ΔGT on the other. In this patient the mAltAB was ~1.25 with increased *NCF1C* in the 5’ region, and normalization of AltAB to 1 after intron 5 (**Figure 4D**). We validated this approach in two NCF1-CGD siblings, in whom the pattern of normalized AltAB for *NCF*1B, NCF1C, and ΨNCF1 was similar, as would be expected given the same parental alleles (**Supplementary Figure 4**).

Having confirmed *NCF1* replacement by *NCF1C* or *NCF1B* in ΔGT patients we looked for the inverse allele in a control population – that is, replacement of *NCF1C* or *NCF1B* by *NCF1*. Examination of ΔGT frequency in 1000G revealed 24 individuals (24/2504; 1%) with ΔGT AltAB<0.4, indicating fewer than 4 copies of ΔGT. In contrast to the rearrangements seen in CGD patients, these individuals had had *NCF1* replacement of a pseudogene. One individual had AltAB = 0.38 for ΔGT, indicating only 2 remaining copies of a pseudogene. SNP analysis across *NCF1B* and *NCF1C* revealed a total loss of *NCF1B* but maintenance of *NCF1C* SNPs, suggesting replacement of *NCF1B* by *NCF1* (**Figure 4E**). Another individual displayed apparent loss of at least one copy of *NCF1C* (**Figure 4F**), although exact determination was difficult due to the lack of *NCF1C* specific variants with normal distribution in the 5’ region.

### Mutation identification

**Figure 5:**
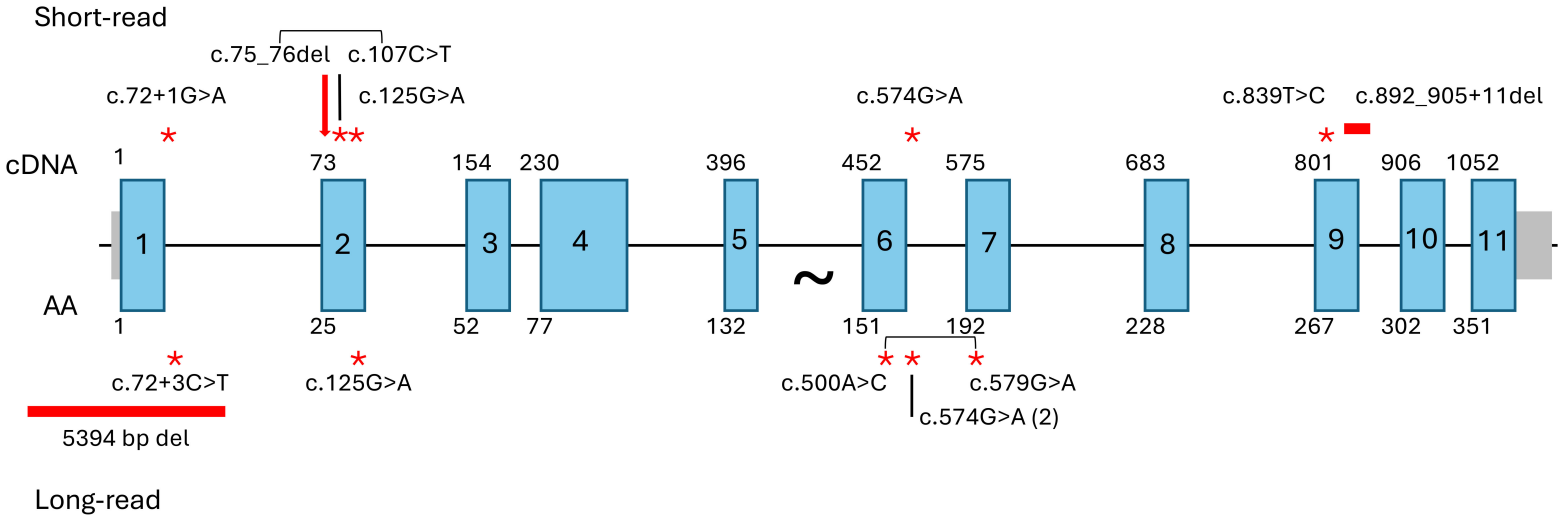
Identified NCF1 mutations. Mutations identified after pseudogene masking of short-read WES/WGS (upper) or long-read WGS (lower). Large arrow indicates ΔGT, point mutations noted by red asterisks, deletions by horizontal red bars. Square brackets indicate allelic variants observed in P5 (short-read) and P8 (long-read).

Having demonstrated AltAB as a valid metric for ΔGT, we used AltAB to screen for non-ΔGT mutations among patients with available short-read sequencing data. Similar to examination of other genes, we first established a variation reference. Analyzing 1000G data with our pipeline identified all variants with an AltAB frequency > 0.08. We determined the number of individuals with each variant as well as minimum, maximum, mean, median and standard deviation of AltAB for each variant; those data were further split by ancestry (**Supplementary Table 1**). Using the variants identified in 1000G, we examined NCF1-CGD patients with 1 or 2 intact GTGT alleles indicating non-ΔGT variations. We considered variants rare or unique among 1000G and the human variation database, Genome Aggregation Database (gnomAD) (15), and predicted deleterious by the bioinformatic algorithm, Combined Annotation Dependent Depletion (16), using a threshold of CADD>20, identifying 6 variants (**Figure 5**, upper; **Table 1**). Each patient with 1 intact GTGT copy had a single mutation, while P1, with 2 GTGT alleles, carried 2 separate mutations; each variant had a frequency approximating 1/6, suggesting that patients were heterozygous for the variants. P1 had two variants, c.72+3G>A at the start of intron 1 and c.125G>A (p.R42Q) within exon 2, both previously reported (14). P2 also carried a previously reported mutation, c.574G>A (p.G192S), which occurs at the last base of exon 6 leading to impaired splicing of the exon (14). P3, P4, and P5 carried novel variants: c.892_905+11del spanning the exon 9/intron 9 boundary, c.839T>C (p.L280P), and c.107C>T (p.S36L), respectively. Three of the variants, c.107C>T, c.125G>A, and c.574G>A, are present in gnomAD at low frequency (~1/100,000) however only c.107C>T occurs in 1000G (n=2). While it is not always possible to phase variants with short-read sequencing, c.107C>T is within 35 bases of ΔGT; using the Integrative Genomics Viewer (17) (IGV), a platform allowing visualization of high throughput sequencing alignments, we determined this variant was allelic with ΔGT (**Supplementary Figure 5**), indicating the variant was not causative of CGD.

### Long-read sequencing

Since we were unable to identify a second mutation in the P5/P6 family, we performed Oxford Nanopore long-read sequencing. With an average read length of 25kb, we confidently spanned the full *NCF1* locus and were able to phase variants across the region. In P6, the niece of P5, we identified a 5394 bp deletion fully encompassing exon 1 and part of intron 1 (chr7:74,770,624-74,776,017), (**Supplementary Figure 6**). Examination of sequencing reads in IGV revealed the large deletion was not allelic with ΔGT (**Supplementary Figure 6**). Using primers spanning the deleted region, the presence of the 5394 bp deletion was confirmed in P5 and the father of P6 (P6-F, an obligate carrier) (**Supplementary Figure 7A**). Additionally, the c.107C>T variant in P5, allelic with ΔGT in P5, was not present in P6 suggesting a commonly inherited large deletion within this family and different ΔGT alleles present in P5 and P6. (**Supplementary Figure 7B**). It is notable that the deceased brother of P5 (uncle of P6) was diagnosed with CGD after *Mycobacterium fortuitum* infection at age 27 (18).

Two additional NCF1-CGD patients were diagnosed using long read sequencing. P7 carried one variant occurring at the last base of exon 6, (c.574G>A; p.G192S/splice) which was non-allelic with ΔGT. P8, with two copies of intact GTGT, had three damaging variants identified, c.72+3G>T at the start of intron 1, c.500A>C encoding p.Y167S, and c.579G>A encoding p.W193*. Examination of reads in IGV revealed that c.500A>C was allelic with the c.579G>A mutation but not the splice mutation (**Supplementary Figure 8**). Lastly, three presumed carriers of non-ΔGT mutations were sequenced. In two cases, a single, known pathogenic mutation was identified which was allelic with intact GTGT; P9, mother of P1, carried one copy of c.125G>A (p.R42Q), while P10 carried c.574G>A (p.G192S/splice). No mutation was identified in P11, the father of an NCF1-CGD patient. It is possible the patient carries a *de novo* mutation on the paternal allele or P11 is a germline mosaic.

## Discussion

While chronic granulomatous disease has been diagnosed in the laboratory for more than 60 years, providing genetic diagnosis for p47^phox^-deficient patients requires specialized techniques performed in select laboratories. We have developed the ability to identify *NCF1* mutations using a bioinformatic pipeline on existing whole exome and whole genome sequence data. By masking the pseudogene sequences and aligning all the reads to *NCF1*, we were able to identify variants within the *NCF1/NCF1B/NCF1C* locus. Using variant vs total read depth provided a variant allele frequency (AltAB) for known variants present in *NCF1B* or *NCF1C* or both (Ψ*NCF1*). Normalizing AltAB in individual patient data to expected variant frequency for these known variants, we demonstrated full or partial replacement of *NCF1* by a pseudogene in 39/42 patients with one patient carrying two non-ΔGT mutations unrelated to pseudogene sequences.

Our data are consistent with previous reports of recombination within the *NCF1/NCF1B/NCF1C* locus, confirming that ΔGT occurs by crossover of *NCF1C/NCF1B* into the *NCF1* locus (8). Most frequently, the entire *NCF1* locus is replaced, however crossovers between exons 2 and 4 or within intron 5 are detected as previously reported (19). The higher observed frequency of *NCF1* replacement by *NCF1C* may be due to the *NCF1/NCF1B/NCF1C* locus organization with *NCF1C* in closer proximity and in reverse orientation to *NCF1* enabling DNA hairpin loop formation and occurrence of crossover events. In many cases of autosomal recessive disease, founder mutations are prominent. While founder mutations may be present in some communities, given the variety of alleles present, it is likely the locus continues to undergo recombination among the *NCF1/NCF1B/NCF1C* alleles. This is supported by the presence of multiple different crossover loci identified here and in the literature (19).

Patients suspected to have CGD are first assessed using assays to quantify the ability of granulocytes to produce reactive oxygen species including DHR or NBT assays. These may be performed by various reference laboratories. Additional testing includes flow cytometric or immunoblotting for NADPH oxidase components (gp91^phox^, p22^phox^, p47^phox^, p67^phox^, and p40^phox^, EROS). In the setting of clinical suspicion plus abnormal functional testing, sequencing may confirm a genetic diagnosis, allowing screening of family members for disease or carrier status. To date, the diagnosis of *NCF1*/p47^phox^ deficiency has been limited to functional testing in affected individuals. There are a handful of laboratories utilizing specialized techniques to enumerate ΔGT copies including Gene-scan (5), droplet digital PCR (ddPCR) (2), and restriction fragment length polymorphism (9), each of which may provide genetic diagnosis for ΔGT. Sequencing of *NCF1* has been reported using primers anchoring on the exon 2 GTGT, allowing identification of non-ΔGT mutations (6, 20, 21), but this too must be performed in a specialized laboratory. As high-throughput sequencing becomes commonplace, the ability to determine *NCF1* variants from high-throughput sequencing permits recognition of NCF1-CGD patients, regardless of clinical presentation. Incorporating our bioinformatic approach would enable identification of individuals presenting later in life, with colitis or Crohn’s disease, or those with previously unappreciated infections, and not limit diagnosis to those children suspected of having a primary immune deficiency.

With 80% of NCF1-CGD patients homozygous for ΔGT, gene correction has become an attractive therapeutic target. Since our data reveal the full replacement of *NCF1* by pseudogene in the majority of NCF1-CGD patients, targeted correction of ΔGT would correct a pseudogene resulting in pseudogene-derived protein expression. Early studies using zinc-finger nucleases demonstrated pseudogene correction was sufficient to restore both p47^phox^ expression and superoxide production (22). Characterizing pseudogene-derived p47^phox^ function, with the associated amino acid differences from *NCF1*-derived p47^phox^, is an important consideration as gene-correction trials are pursued. Correction of one or both pseudogenes would likely result in a protein containing p.R90H, reported to cause an early-onset interferonopathy (23), or a lupus-like disease in mice (24). The 12 healthy individuals from 1000G, who have only p.R90H, suggest additional factors may play a role in the immune dysregulation reported for this variant. Additionally, pseudogene correction that restores myeloid cell function may also benefit patients with non-ΔGT mutations.

Here we have developed a technique using standard, high throughput sequencing to establish the genetic diagnosis of NCF1/p47^phox^ CGD. Using this bioinformatic pipeline does not require specialized instrumentation, techniques, or the need for resequencing and may be performed on historic high-throughput sequence data. It is important to note that, depending on the sequencing platform used, this method may not establish whether the mutation occurs within *NCF1* or one of the pseudogenes. Long-read sequencing spanning the full length of *NCF1* allows phasing of identified variants with ΔGT or GTGT at the start of exon 2 and other pseudogene-specific SNPs; this is not possible with short-read sequencing. This approach should always be accompanied by functional assays demonstrating abnormal neutrophil respiratory burst and loss of p47^phox^ protein. Additionally, the diagnosis of NCF1-CGD in older children and adults is not uncommon; adult cases have been diagnosed presenting with pneumonia (25–28) or in the setting of colitis (29, 30). These cases suggest a broader use for the pipeline beyond pediatric immune deficiency patients including individuals with recurrent infections, *Aspergillus fumigatus* pneumonia, Crohn’s disease, or other forms of colitis. Providing genetic and clinical diagnoses in these settings allows proper antimicrobial treatment and ongoing prophylaxis for the patients and the ability to screen at-risk family members for *NCF1* mutation status. This method should be modifiable for other gene/pseudogene combinations which inhibit standard sequencing diagnosis such as *IKBKG*/NEMO deficiency, associated with immune deficiency with or without ectodermal dysplasia.

## Data Availability

The datasets presented in this study can be found in online repositories. The names of the repository/repositories and accession number(s) can be found in the article/**Supplementary Material**.
