# Supplementary material for: Reliable genetic diagnosis of NCF1 (p47phox)-deficient chronic granulomatous disease using high-throughput sequencing
Source: Front Immunol. 2025 Aug 18;16:1640496. doi: 10.3389/fimmu.2025.1640496 (PMC12418599; doi:10.3389/fimmu.2025.1640496)
Supplement: Supplementary file 2 [file DataSheet1.pdf]

Supplemental Materials, Hsu et al, *NCF1* diagnosis by high-throughput sequencing

Supplemental Table 1. 1000 Genomes NCF1 variants.xlsx
AltAB data for each *NCF1* variant in 1000 Genomes cohort. Available as separate download file

Supplemental Table 2. Neutrophil functional assessment NCF1-CGD patients

Patient ID	Disease	Basal O_2^- [0.00-4.80]	PMA-stimulated O_2^- [135.20-348.15]	Basal DHR MFI [49-111]	PMA DHR MFI [9475 - 15845]
P1[A]	NCF1-CGD	1.27±0.89 (6)	**3.02 ± 1.10 (6)**	117 ± 28 (9)	**796 ± 457 (9)**
P2	NCF1-CGD	0.85 ± 0.03 (2)	**2.60 ± 0.08 (2)**	90 ± 21 (2)	**344 ± 167 (2)**
P3	NCF1-CGD	1.16 ± 0.36 (4)	**2.38 ± 0.75 (4)**	121 ± 20 (2)	**1211 ± 113 (2)**
P4	NCF1-CGD	0.67 ± 0.09 (2)	**2.50 ± 0.42 (2)**	111 ± 7 (2)	**602 ± 44 (2)**
P5[B]	NCF1-CGD	0.98 ± 0.46 (10)	**2.15 ± 0.64 (10)**	121 ± 32 (5)	**371 ± 49 (5)**
P6[B]	NCF1-CGD	1.41 ± 0.73 (10)	**3.42 ± 0.97 (10)**	109 ± 1 (2)	**1117 ± 257 (2)**
P7	NCF1-CGD	0.62 ± 0.05 (3)	**1.66 ± 0.63 (3)**	126 ± 50 (3)	**549 ± 234 (3)**
P8	NCF1-CGD	0.73 (1)	**2.43 (1)**	82 (1)	**1371 (1)**
P9[A]	NCF1 carrier	1.82 (1)	199.7 (1)	121 ± 1 (2)	13729 ± 3336 (2)
P10	NCF1 carrier	1.10 (1)	184.60 (1)	117 (1)	13764 (1)
P11	presumed NCF1 carrier	3.03 ± 1.74 (2)	188.98 ± 11.65 (2)	n.d.	n.d.
P12[C]	NCF1-CGD	1.75 ± 0.84 (2)	**3.58 ± 0.83 (2)**	62 ± 2 (2)	**403 ± 6 (2)**
P13[C]	NCF1-CGD	0.94 ± 0.27 (5)	**3.30 ± 0.37 (5)**	70 ± 13 (4)	**294 ± 61 (4)**
P14	NCF1-CGD	1.64 ± 0.93 (3)	**3.14 ± 1.05 (3)**	78 ± 9 (2)	**254 ± 66 (2)**
P15	NCF1-CGD	0.54 ± 0.09 (3)	**2.01 ± 0.15 (3)**	79 ± 7 (3)	**384 ± 74 (3)**
P16	NCF1-CGD	1.69 ± 1.41 (3)	**3.21 ± 1.54 (3)**	99 ± 41 (2)	**268 ± 80 (2)**
P17	NCF1-CGD	1.17 ± 0.42 (4)	**2.76 ± 0.31 (4)**	77 ± 9 (3)	**412 ± 22 (3)**
P18[D]	NCF1-CGD	1.91 ± 1.08 (4)	**4.09 ± 1.20 (4)**	70 ± 11 (2)	**1705 ± 217 (2)**
P19[D]	NCF1-CGD	0.93 ± 0.16 (2)	**3.65 ± 0.53 (2)**	94 ± 21 (5)	**1544 ± 749 (5)**
P20[E]	NCF1-CGD	0.88 ± 0.48 (4)	**2.85 ± 0.70 (4)**	103 ± 24 (4)	**452 ± 83 (4)**
P21[E]	NCF1-CGD	1.57 ± 0.91 (3)	**2.87 ± 1.15 (3)**	88 ± 20 (3)	**579 ± 99 (3)**
P22	NCF1-CGD	0.61 ± 0.16 (3)	**2.58 ± 0.23 (3)**	91 ± 16 (2)	**303 ± 33 (2)**
P23	NCF1-CGD	0.67 ± 0.27 (3)	**3.45 ± 0.44 (3)**	98 ± 25 (5)	**1295 ± 442 (5)**
P24	NCF1-CGD	0.94 ± 0.06 (3)	**3.86 ± 0.30 (3)**	89 ± 25 (4)	**1328 ± 258 (4)**
P25	NCF1-CGD	1.25 ± 0.50 (2)	**2.95 ± 0.30 (2)**	81 ± 30 (2)	**701 ± 516 (2)**
P26[F]	NCF1-CGD	1.00 ± 0.41 (9)	**2.83 ± 1.18 (9)**	125 ± 7 (2)	**585 ± 165 (2)**
P27[F]	NCF1-CGD	0.83 ± 0.18 (4)	**2.13 ± 1.12 (4)**	59 ± 10 (4)	**413 ± 142 (4)**

P28	NCF1-CGD	0.56 ± 0.16 (3)	**1.47 ± 0.55 (3)**	101 ± 12 (3)	**361 ± 151 (3)**
P29	NCF1-CGD	0.82 (1)	**2.62 (1)**	154 (1)	**797 (1)**
P30	NCF1-CGD	0.67 ± 0.04 (3)	**2.66 ± 0.20 (3)**	122 ± 19 (3)	**1006 ± 207 (3)**
P31	NCF1-CGD	0.80 ± 0.16 (2)	**2.56 ± 0.77 (2)**	70 ± 14 (3)	**229 ± 56 (3)**
P32	NCF1-CGD	1.25 ± 0.67 (4)	**2.82 ± 1.58 (4)**	83 ± 17 (3)	**322 ± 73 (3)**
P33	NCF1-CGD	1.13 ± 0.48 (3)	**3.40 ± 0.31 (3)**	78 ± 2 (2)	**911 ± 179 (2)**
P34	NCF1-CGD	1.66 ± 0.86 (2)	**3.44 ± 0.32 (2)**	88 (1)	**762 (1)**
P35	NCF1-CGD	0.73 ± 0.16 (2)	**2.61 ± 0.30 (2)**	90 ± 2 (2)	**646 ± 259 (2)**
P36	NCF1-CGD	1.00 ± 0.35 (6)	**2.82 ± 0.79 (6)**	75 ± 7 (3)	**237 ± 18 (3)**
P37	NCF1-CGD	1.18 ± 0.50 (2)	**3.91 ± 0.58 (2)**	108 ± 11 (3)	**829 ± 259 (3)**
P38	NCF1-CGD	0.94 ± 0.06 (3)	**3.38 ± 0.19 (3)**	77 ± 4 (2)	**478 ± 166 (2)**
P39	NCF1-CGD	0.65 (1)	**2.16 (1)**	114 (1)	**324 (1)**
P40	NCF1-CGD	1.31 (1)	**3.97 (1)**	103 (1)	**560 (1)**
P41	NCF1-CGD	0.60 (1)	**2.82 (1)**	88 (1)	**1469 (1)**
P42	NCF1-CGD	0.73 ± 0.16 (4)	**2.87 ± 0.35 (4)**	91 ± 7 (2)	**451 ± 68 (2)**
P43	NCF1-CGD	0.47 (1)	**2.66 (1)**	100 (1)	**510 (1)**
P44	NCF1-CGD	0.38 (1)	**2.42 (1)**	58 (1)	**322 (1)**
P45	NCF1-CGD	1.22 ± 0.51 (7)	**3.20 ± 0.49 (7)**	70 ± 15 (2)	**593 ± 200 (2)**
P46	NCF1-CGD	1.12 (1)	**3.17 (1)**	72 (1)	**446 (1)**
P47	NCF1-CGD	1.60 ± 0.89 (2)	**3.24 ± 0.70 (2)**	88 ± 19 (3)	**546 ± 61 (3)**
P48	NCF1-CGD	0.92 ± 0.22 (3)	**3.37 ± 0.83 (3)**	92 ± 27 (5)	**529 ± 206 (5)**

Neutrophil functional studies for NCF1 cohort. Basal and PMA-induced superoxide (O_2^-) and mean fluorescence intensity (MFI) for flow cytometric determination of dihydrorhodamine-123 (DHR) reduction. Patients are numbered as in Table 1. Related individuals are denoted by matching colors in Patient ID. Superoxide production expressed in nmoles/10^6 cells/hr; DHR MFI expressed in arbitrary units. Reference ranges shown in square brackets, number in parentheses indicate number of tests per patient. Values below the reference range shown in **bold**.

Supplemental Table 3. *NCF1/NCF1B/NCF1C* variant table

chr7 position	*NCF1* Ref	*NCF1B* Alt	*NCF1C* Alt	1000G occurrence*	Assigned Group
74775097	G		A	2339	*NCF1C*
74775774	G	A		246	*NCF1B*
74775890	T	C	C	2504	*ΨNCF1*
74775992[&]	G	A		2487	*NCF1B*
74776509[#]	A	C	C	2504	*ΨNCF1*
74777145	C	T	T	2504	*ΨNCF1*
74777270	T	:	:	10	*ΨNCF1*
74777361[#]	T	C	C	8	*ΨNCF1*
74777569	C	G	G	2504	*ΨNCF1*
74777644	G	A	A	1	*ΨNCF1*
74778377	C	T	T	2504	*ΨNCF1*
74778595	G		A	1603	*NCF1C*
74778644	C	T	T	2504	*ΨNCF1*
74778736[#]	A	G	G	2504	*ΨNCF1*
74778776	G	A	A	2504	*ΨNCF1*
74779197	C		T	317	*NCF1C*
74779221	C	A		2491	*NCF1B*
74779296	G	A	A	2502	*ΨNCF1*
74779322	A	G		2494	*NCF1B*
74779372	C	T		2479	*NCF1B*
74779691[&]	C	T		2478	*NCF1B*
74780198	G	C	C	2504	*ΨNCF1*
74780244[&]	G	A		2490	*NCF1B*
74781407[#]	T	C	C	2504	*ΨNCF1*
74781580[#]	T	C	C	2504	*ΨNCF1*
74781655	C		T	2233	*NCF1C*
74781714	G	A	A	2504	*ΨNCF1*
74781743	A	G	G	2504	*ΨNCF1*
74781855[&]	C	T		2502	*NCF1B*
74781899[#]	T	C	C	2504	*ΨNCF1*
74781945[#]	C	T	T	2504	*ΨNCF1*
74781952	A	G	G	2504	*ΨNCF1*
74782003	G	A	A	2504	*ΨNCF1*
74782019	T	C	C	2504	*ΨNCF1*
74782308	G	A		1164	*NCF1B*
74782482	A	G		2504	*NCF1B*

74782613#	A	G	G	2504	*ΨNCF1*
74782668	C	T	T	2504	*ΨNCF1*
74782693	G		A	406	*NCF1C*
74782790&	G	A		2502	*NCF1B*
74782983&	A		G	2504	*NCF1C*
74783045	A	G	G	2504	*ΨNCF1*
74783147	T	G	G	2504	*ΨNCF1*
74783165&	G		A	2503	*NCF1C*
74783215	G	C	C	2504	*ΨNCF1*
74783571	G	A	A	2504	*ΨNCF1*
74783765	A	T	G	98	*ΨNCF1*
74783840	G	C	C	2503	*ΨNCF1*
74784190	A	G	G	2503	*ΨNCF1*
74784709	T	C		2504	*NCF1B*
74785264&	C	A		2478	*NCF1B*
74785449	A	G	G	1	*ΨNCF1*
74785617	T	C	C	2504	*ΨNCF1*
74785652	A	G		2504	*NCF1B*
74785664	T	C	C	2504	*ΨNCF1*
74785746	G	T	T	2504	*ΨNCF1*
74785798	A	G	G	2504	*ΨNCF1*
74785822	G	A	A	2504	*ΨNCF1*
74786081	A		T	2201	*NCF1C*
74786214	G		A	407	*NCF1C*
74786460	A	G	G	2504	*ΨNCF1*
74786577	A	G	G	2504	*ΨNCF1*
74786646&	T	A		2502	*NCF1B*
74787224	T	G	G	2504	*ΨNCF1*
74787344	A	G	G	2504	*ΨNCF1*
74787450	C	T	T	2504	*ΨNCF1*
74787561	C	T	T	2504	*ΨNCF1*
74787566	A	G	G	2504	*ΨNCF1*
74787585	C	A	A	2453	*ΨNCF1*
74788008&	C		T	2503	*NCF1C*
74788032&	A		G	2504	*NCF1C*
74788213&	C		T	2491	*NCF1C*
74788320#	G	C	C	2504	*ΨNCF1*
74788576&	C	T		2503	*NCF1B*
74788589&	C		T	2504	*NCF1C*
74788744	A	G	G	2504	*ΨNCF1*

74788777[&]	T		C	2501	NCF1C
74788845	T	G	G	2499	*ΨNCF1*
74788952	C		A	2498	NCF1C
74789215	T		C	2498	NCF1C
74789492[^]	C		T	2504	NCF1C
74789497[^]	T		A	2504	NCF1C

* - Number of individuals in 1000G with variant (n = 2504)

ΨNCF1 - Occurs in both *NCF1B/NCF1C*

& - variants in one pseudogene with normal 1000G distribution and median AltAB ~0.33

^ - variants in one pseudogene with normal 1000G distribution but median AltAB <0.3

# - variants in *ΨNCF1* with median AltAB ~1.0

Supplemental Figure 1. AltAB for *ΨNCF1* variants in 1000G

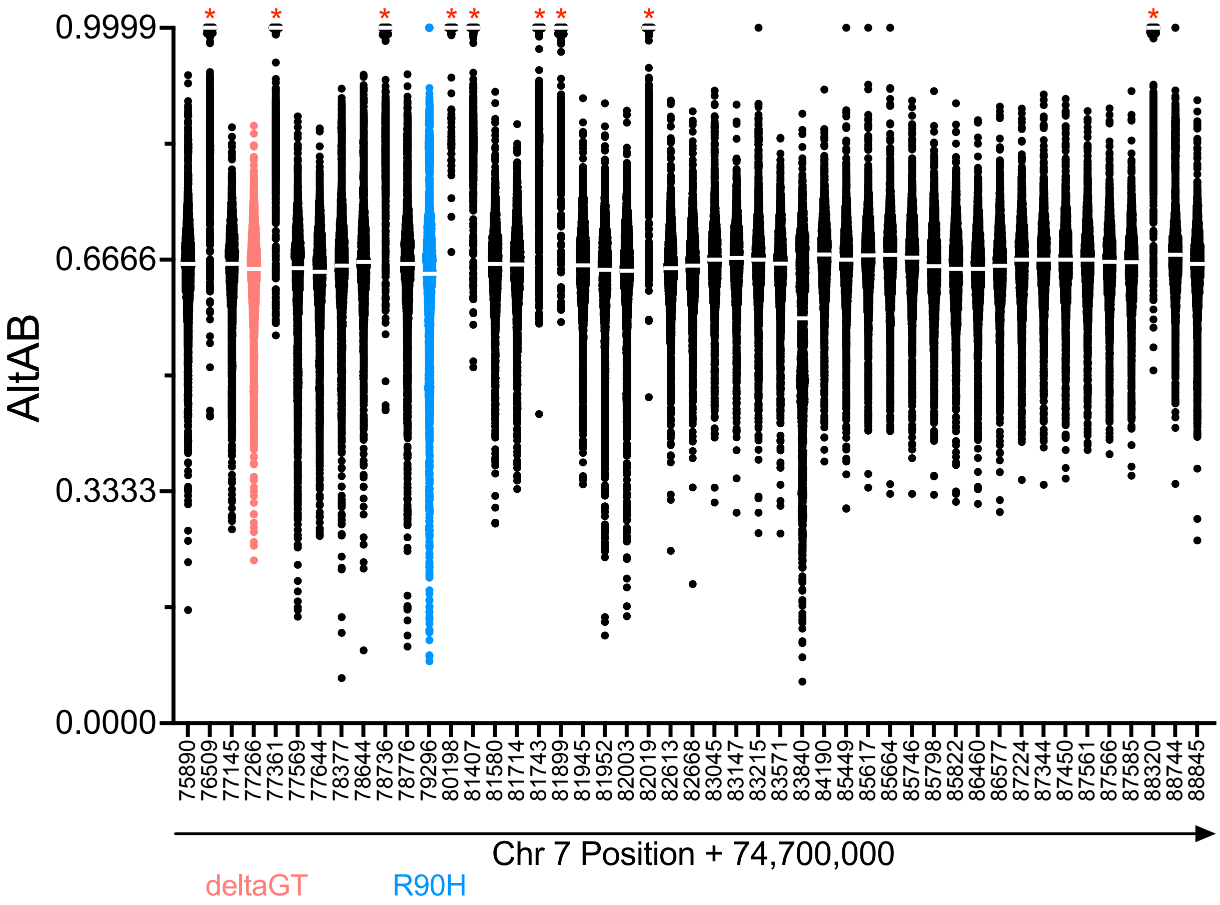

White bar is median AltAB for each variant.
* indicates variants with median AltAB = 1.0 (n=9). Chromosome 7 location for each variant is 74,700,000 + listed position.
Location of c.75_76del causing ΔGT is shown in pink, p.R90H variant shown in blue.

Supplemental Figure 2. AltAB of *NCF1C* referenced variants from 1000G

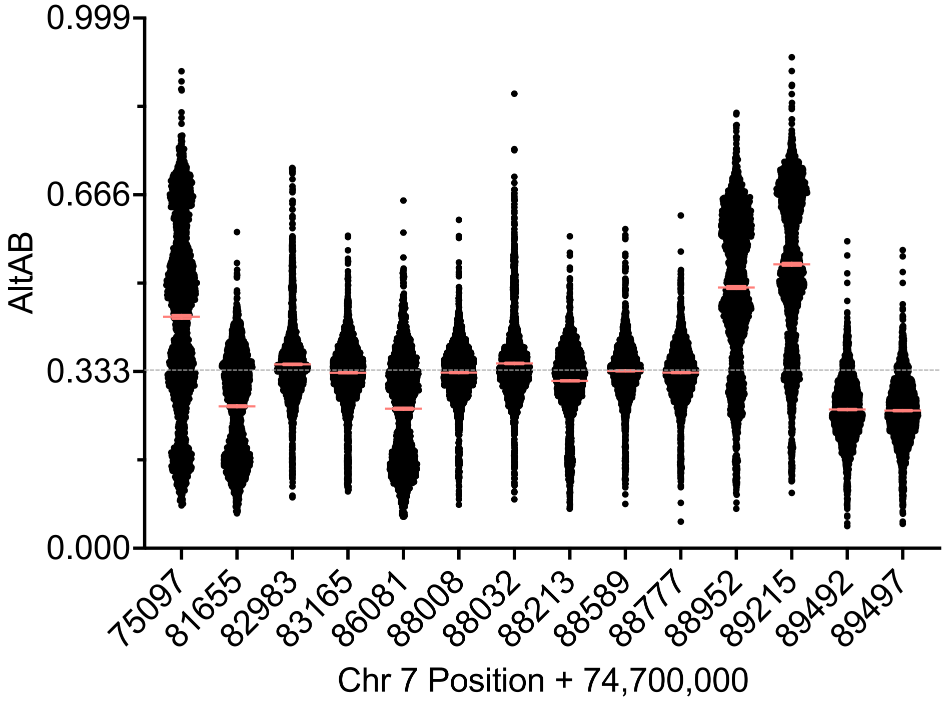

Median for each variant shown as red line, grey line denotes AltAB = 0.33.

Supplemental Figure 3. AltAB of *NCF1B* referenced variants from 1000G

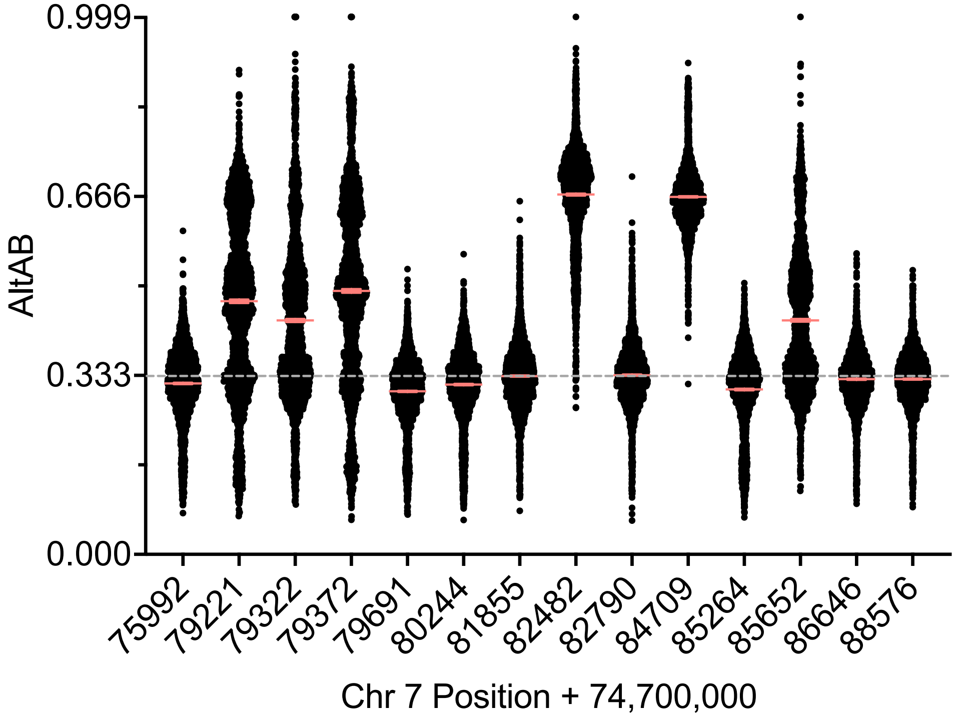

AltAB for 1000G on subset of variants referenced as *NCF1B*. Median for each variant shown as red line, grey line denotes AltAB = 0.33.

Supplemental Figure 4. Normalized AltAB for affected siblings

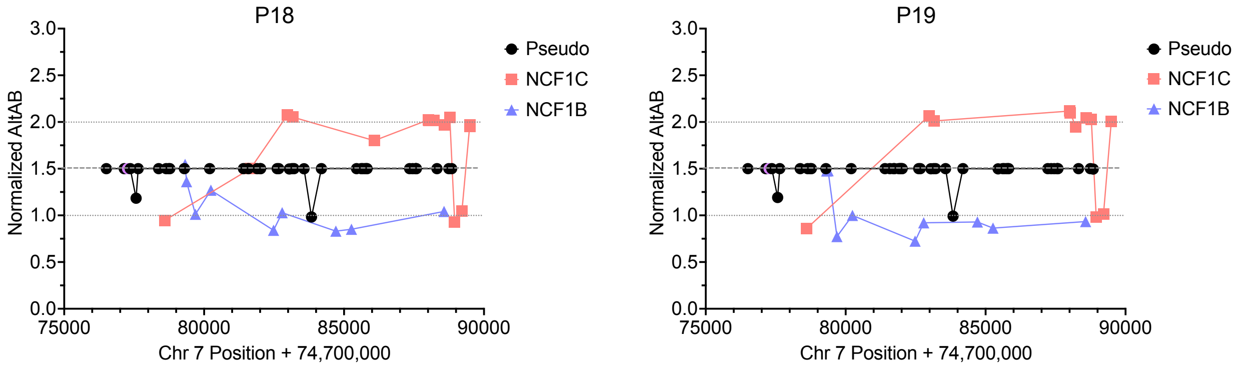

Plot of normalized AltAB for affected siblings, P18 (left) and P19 (right).

Supplemental Figure 5. P5 variant c.107C>T is allelic with ΔGT

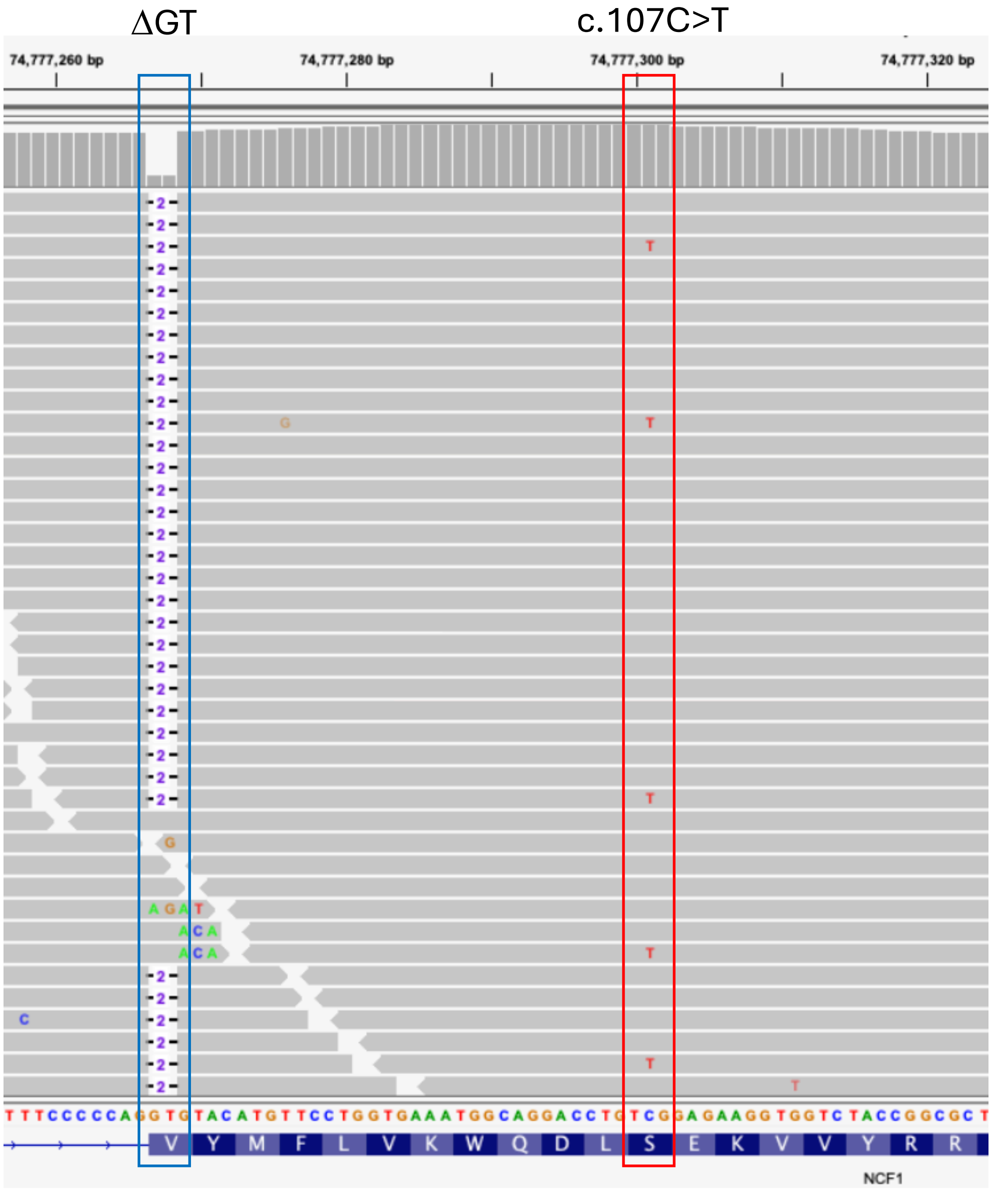

Integrative Genomics Viewer (IGV) image displaying whole exome sequence reads for P5 spanning *NCF1* exon 2. The assembled reads demonstrate c.107C>T (red box) occurs on the same reads as ΔGT (blue box) indicating they are allelic.

Supplemental Figure 6. Large deletion identified in P6

A.

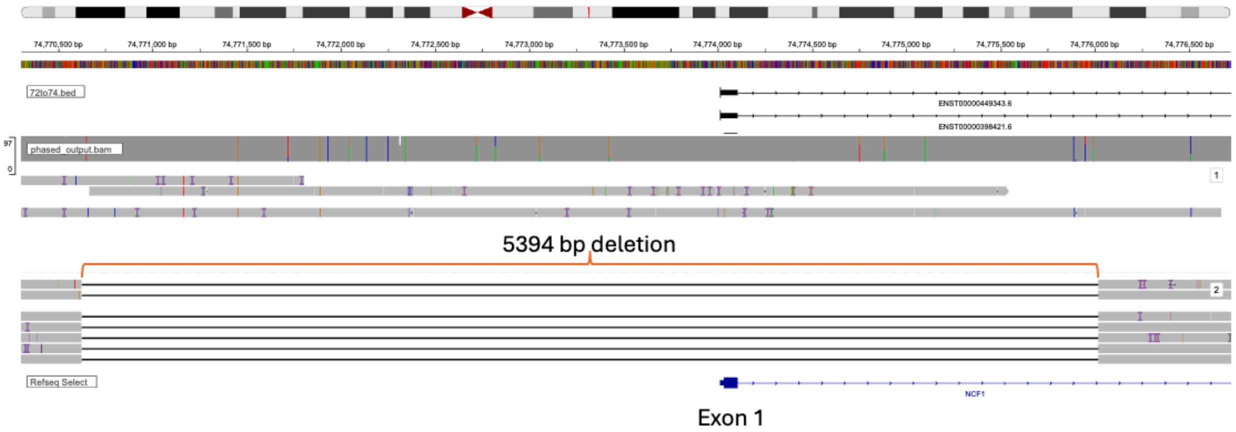

B.

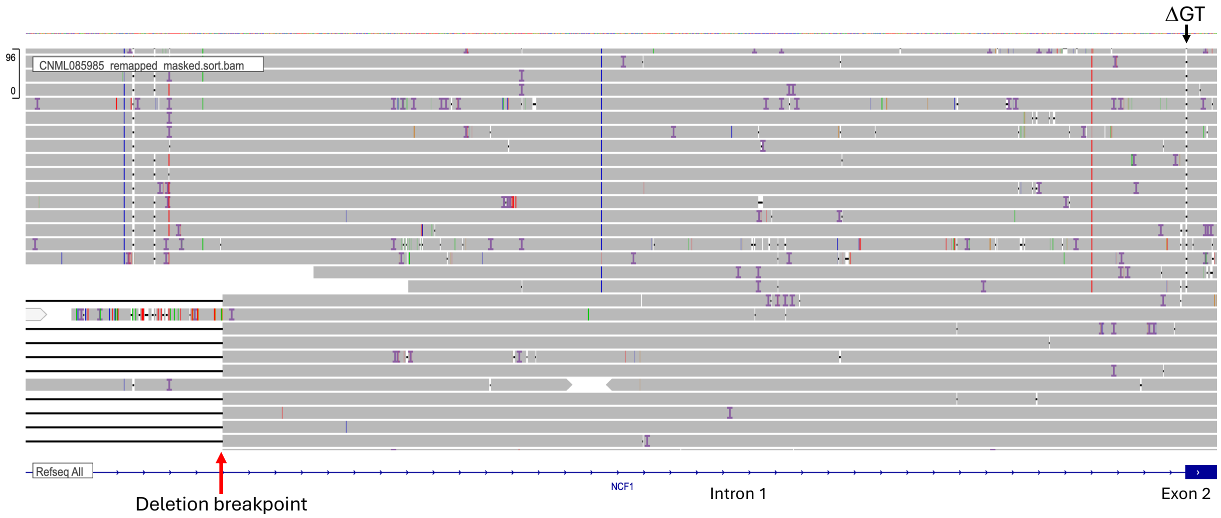

Integrative Genomics Viewer (IGV) image for P6 demonstrating 5394 base pair (bp) deletion. A. Full deletion spanning exon 1 and portions of proximal promoter and intron 1 noted by red bracket. B. Individual sequence reads demonstrates the 3’ breakpoint of the 5394 bp deletion from P6 (red lower arrow) are not allelic with ΔGT at the start of exon 2 (black upper arrow).

Supplemental Figure 7. Confirmation of large deletion in P5/P6 family

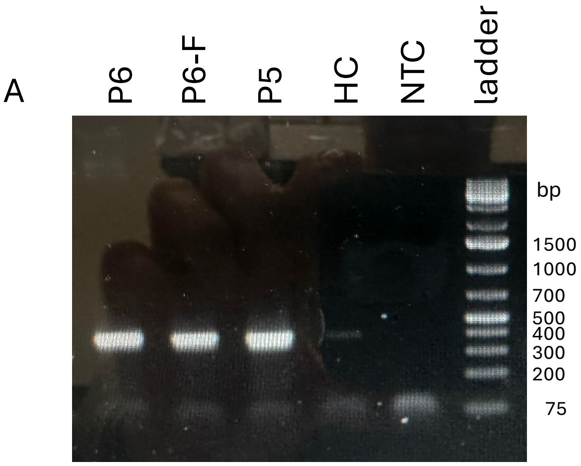

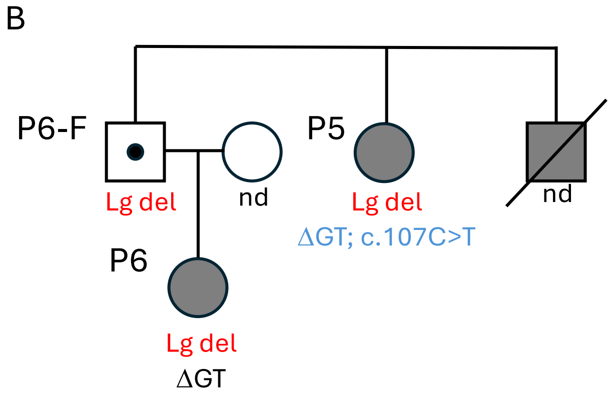

A. PCR using primers spanning 5394 bp deletion identified by Oxford Nanopore whole genome sequencing. HC – healthy control, NTC – no template control, ladder – GeneRuler 1 kb DNA ladder, sizes (base pairs - bp) on right. B. Pedigree of P5/P6 family. Identified mutations are listed below each individual's symbol: commonly inherited large deletion (red) and two distinct ΔGT alleles in P5 (allelic ΔGT and c.107C>T indicated in blue) and P6 (ΔGT in black); nd = not tested. Circles = females, squares = males, filled symbol = affected, small circle inside symbol = unaffected mutation carrier.

Supplemental Figure 8. Phasing of variants observed in P8

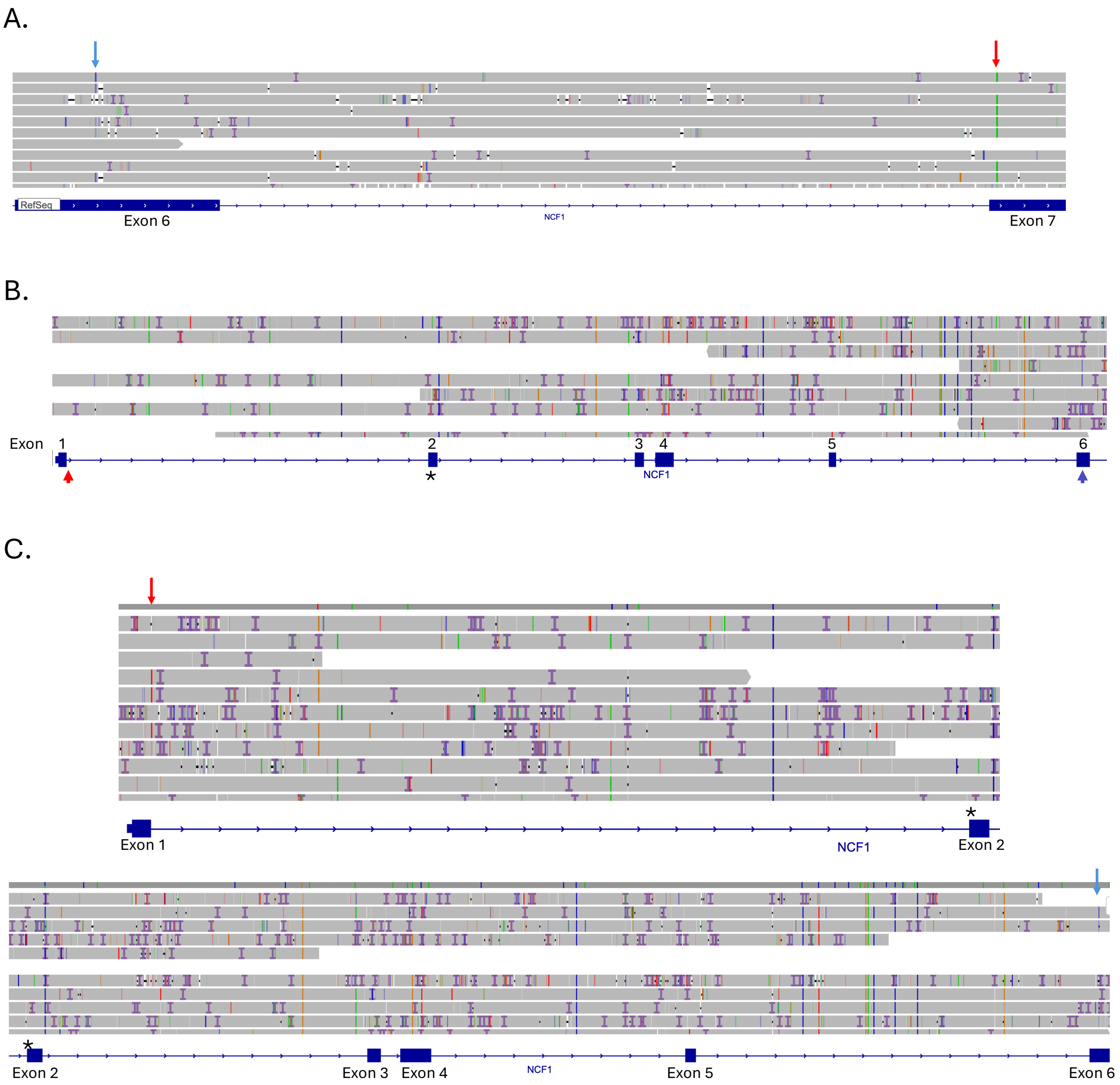

Integrative Genomics Viewer (IGV) image for P8 demonstrating two mutant alleles, one containing c.500G>A and c.579G>A and a second containing a splice mutation at the start of intron 1. A. Sequencing reads spanning exons 6 and 7 showing the same reads contain both c.500A>C (blue arrow, blue lines within reads) and c.579G>A (red arrow, green lines within reads). B. Sequencing reads spanning exons 1 through 6 showing reads with c.72+3G>A (red arrow) do not contain c.500G>A (blue arrow) indicating the two variants are not allelic. C. Reads containing either mutation (c.72+3G>A, red arrow, top; c.500G>A, blue arrow, bottom) do not contain ΔGT (black asterisk)
